# Serological evidence of zoonotic filovirus exposure among bushmeat hunters in Guinea

**DOI:** 10.1038/s41467-024-48587-5

**Published:** 2024-05-16

**Authors:** Joseph Akoi Boré, Joseph W. S. Timothy, Tom Tipton, Ifono Kekoura, Yper Hall, Grace Hood, Stephanie Longet, Kimberly Fornace, Millimono S. Lucien, Sarah Katarina Fehling, Beatrice K. Koivogui, Si’Ana A. Coggins, Eric D. Laing, Christopher C. Broder, N’ Faly Magassouba, Thomas Strecker, Jeremy Rossman, Kader Konde, Miles W. Carroll

**Affiliations:** 1 Ministère de la Santé et de l’hygiène publique, Conakry, Guinea; 2 Faulty of Infectious & Tropical Diseases, London School of Hygiene Tropical Medicine, London, UK; 3 Centre for Human Genetics & Pandemic Sciences Inst, University of Oxford, Oxford, UK; 4 UK Health Security Agency, Porton Down, UK; 5 Saw Swee Hock School of Public Health, National University of Singapore, Singapore, Singapore; 6 Institute of Virology, Philipps University, Marburg, Germany; 7 Department of Microbiology and Immunology, Uniformed Services University, MD, USA; 8 School of Bioscience, University of Kent, Canterbury, UK; 9 Centre for Training and Research on Priority Diseases including Malaria in Guinea, Conakry, Guinea

**Keywords:** Ebola virus, Viral infection

## Abstract

Human Ebola virus (EBOV) outbreaks caused by persistent EBOV infection raises questions on the role of zoonotic spillover in filovirus epidemiology. To characterise filovirus zoonotic exposure, we collected cross-sectional serum samples from bushmeat hunters (*n* = 498) in Macenta Prefecture Guinea, adjacent to the index site of the 2013 EBOV-Makona spillover event. We identified distinct immune signatures (20/498, 4.0%) to multiple EBOV antigens (GP, NP, VP40) using stepwise ELISA and Western blot analysis and, live EBOV neutralisation (5/20; 25%). Using comparative serological data from PCR-confirmed survivors of the 2013-2016 EBOV outbreak, we demonstrated that most signatures (15/20) were not plausibly explained by prior EBOV-Makona exposure. Subsequent data-driven modelling of EBOV immunological outcomes to remote-sensing environmental data also revealed consistent associations with intact closed canopy forest. Together our findings suggest exposure to other closely related filoviruses prior to the 2013-2016 West Africa epidemic and highlight future surveillance priorities.

## Abstract (web summary)

In a serological analysis, Bore et al. utilise serum samples, collected from a cohort of individuals associated with bushmeat hunting and butchering, in the forested region of Guinea, a region close to the epicentre of the 2013–2016 West Africa Ebola virus disease epidemic.

## Introduction

In the past decade, repeated *Ebolavirus* outbreaks in West and Central Africa have resulted in public health emergencies of increasing frequency. These events include two outbreaks designated by the World Health Organization (WHO) as public health emergencies of international concern (PHEIC). Both outbreaks were caused by the pathogen Zaire Ebolavirus (EBOV) from the *Ebolavirus* genus (family *Filoviridae*). EBOV is transmitted through close human-to-human contact, often via exposure to bodily fluids of symptomatic or deceased patients following initial spillover into humans from sylvatic reservoirs^1^. Infection typically results in multi-system Ebola virus disease (EVD) leading to hypovolemic shock and death with a high case fatality rate (CFR). Recent studies have also demonstrated that mildly symptomatic forms of disease are an uncommon yet bona fide component of EVD and are sufficient to generate a detectable antibody response^2^.

An unresolved question persisting across all *Ebolavirus* species is their natural history prior to establishment in human populations. Human outbreaks typically arise following spillover of the pathogen from a natural reservoir or intermediate host. Historical outbreak investigations provide convincing evidence of these occurrences, with clear epidemiological links between suspected index cases and exposure to potentially infected animals^3^. Bats remain widely postulated as the principal upstream reservoir of *Ebolaviruses*. Evidence in support of this hypothesis includes repeated isolation of RNA or anti-EBOV antibodies from an ever-growing number of bat species^4^, immunotolerance among *Chiropteran* families to highly pathogenic RNA viruses^5^ and recent isolation of a novel *Ebolavirus* genome (Bombali virus; BOMV) from healthy bats in Sierra Leone^6^.

The 2013-2016 West Africa epidemic was the largest known human EBOV outbreak leading to at least 28,646 cases and 11,323 deaths^7^. Even following two subsequent outbreaks in Nzérékoré Prefecture (Forested Guinea), caused by reactivation of persistent virus in previously infected cases, this remains the only known zoonotic *Ebolavirus* outbreak in West Africa, aside from a single non-fatal case of Taï Forest virus in Côte d’Ivoire in 1994.

The 2013 outbreak began in Meliandou, Guéckédou Prefecture (Guinée Forestière) and was linked, though not conclusively, to interaction of the index case with insectivorous bats^8^. Subsequent ecological studies failed to identify evidence of EBOV near Meliandou^9^. Further investigations did, however, result in detection of EBOV RNA fragments from *Miniopterus inflatus* bats near the Liberia-Guinea border area and RNA fragments of BOMV from bats (*Mops condylurus)* at multiple sites in the adjacent Nzérékoré Prefecture^10^. Recently, the first case of Marburg virus (MARV), also a filovirus, has also been reported in West Africa. The index case was also identified in Guéckédou Prefecture and zoonotic exposure the probable aetiology. Unlike *Ebolaviruses*, more information on the natural history of MARV has been obtained. Asymptomatic transmission among cave dwelling *Rousettus aegypticatus* bats, pulsed virus shedding and human spillover events epidemiologically linked to contact with bat reservoirs create a plausible link between the ecological stages of spillover^11^. Importantly, *Rousettus aegypticatus* are prevalent in West Africa and MARV was recently isolated from these bats in Sierra Leone^12^.

Whilst the ultimate zoonotic origin and/or reservoir host(s) of *Ebolaviruses* remains to be elucidated, the continued outbreaks of EBOV in the Democratic Republic of Congo (DRC) and Guinea have challenged the notion of a single type of spillover model from an animal reservoir. Here these outbreaks were both caused by persistent viral reactivation, with EBOV dormancy in the Guinean example persisting for over 5 years^13^. Recent publication of a retrospective investigation from Meliandou also identified a speculative though plausible pathway through which EBOV may have been introduced into Guinea via migration of previously infected individuals^14^. With the emerging realisation of both EBOV latency and MARV as a public health threat in West Africa, it is of increasing importance to delineate the natural history, population exposure or spillover propensity of endemic filoviruses.

An effective tool to overcome the challenge of disentangling the ecological dynamics of *Ebolaviruses*, is the application of seroepidemiology at the human-wildlife interface. Historical seroprevalence studies across Africa previously attempted to delineate the ecological niche of *Ebolaviruses*. These studies showed vastly different estimates of exposure, often ascribed to variations in assay choice and analysis procedures^15^. Efforts were also confounded by the potential of related filoviruses influencing immune characteristics in EBOV-endemic areas^16^. Serological cross-reactivity across *Filoviridae* species is well-characterised and the recent identification of BOMV coupled to the serendipitous discovery of other *Ebolaviruses* (e.g. Taï Forest virus) highlight the knowledge gaps in the Filoviridae virosphere^17^. Recent longitudinal studies among EBOV survivors in West Africa, however, have strengthened our understanding of serological responses to natural EBOV infection and provide a platform to improve seroepidemiological approaches. These studies demonstrate persistent and slow-decaying B-cell mediated antibody responses, supporting previous observations of anti-EBOV immunoglobulin-G (IgG) persistence and neutralisation over 40-years after recovery from infection^18–20^. Exposure to *Ebolaviruses*, therefore, generates persistent immune signatures that are amenable for seroepidemiological investigations, however the antigenic cross-reactivity between virus species necessitates the use of a multi-faceted serological approach based on well characterised assays.

To understand the potential for zoonotic EBOV spillover in Guinea we undertook a multi-stage immunological analysis of serum purposively collected from bushmeat hunters and their family members, a population with suspected high potential risk of exposure to zoonotic pathogens like EBOV^3,21^. We conducted the study in Macenta Prefecture, Guinea. This location was selected due to its intersection with suspected filovirus index sites (Guéckédou) and areas with evidence of *Ebolavirus* occurrence in bat populations.

## Results

### Serological analysis

**Fig. 1:**
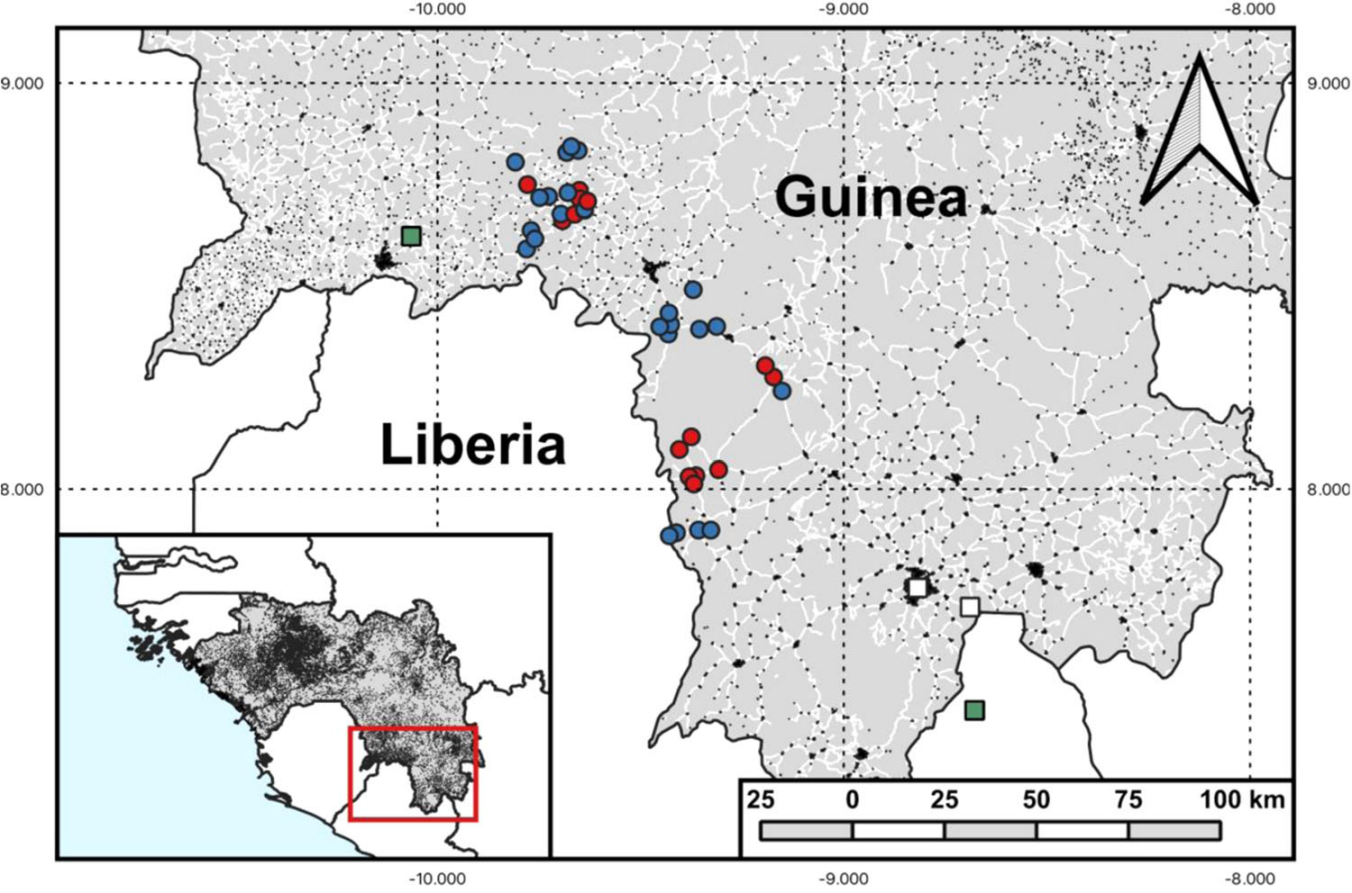
Sampling locations of villages in Macenta Prefecture, Guinea. Points represent centroids of sample villages coloured red if EBOV-affected (PCR-confirmed EBOV 2013–16) and blue if unaffected. Green squares represent approximate locations of EBOV index cases or EBOV RNA fragment isolation from bats (*Miniopterus inflatus)*. White squares show locations of Bombali virus RNA fragment isolation from bats (*Mops condylurus)*. Black marks indicate intermediate and large settlements as delineated by GRID3 as indicators of density. White lines indicate non-minor roads suitable for motorised vehicles extracted from OpenStreetMap to indicate accessibility. Figure generated using QGIS software.

We interviewed and collected serum samples from 499 people who actively engage in bushmeat hunting and their family members from 38 villages in Macenta prefecture of Guinea (Fig. 1). The median village population based on health facility records was 536 (range 52-3901, IQR 187-1800) and the mean number of participants sampled per village was 13 (range 2–35, 95% CI 11-15). Among sampled villages, 14 (36.8%) reported PCR-confirmed EBOV cases during the 2013–16 outbreak. The remaining 24 villages had no suspected or confirmed EBOV cases. Absence of cases was validated through in-depth key informant interviews with local healthcare workers, village leaders and community health agents. Of participants sampled across all villages, 280 were male (43.9%) and the median age was 39 (range 18–90, IQR 28–48). There were 276 bushmeat hunters among the 499 sampled (55.3%), the majority of whom were male (271; 98.2%). The remaining participants were either the marriage partner or the next closest household relative to the hunter (214 female, 96.0%).

**Fig. 2:**
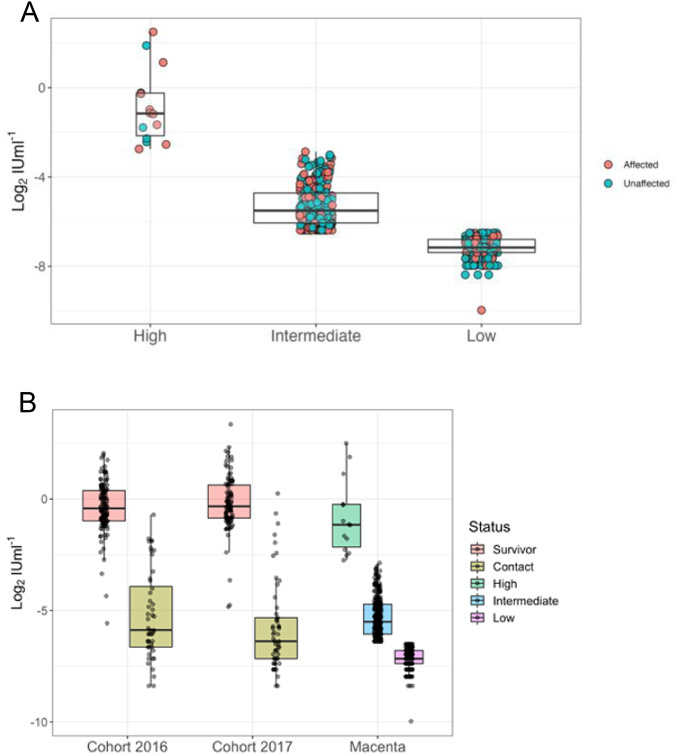
Individual-level responses of Macenta and EVD survivor serum samples using EBOV-GP ELISA. Point colours indicate village status of participant (blue = unaffected, red = affected) and groups are based on fit of latent profile analysis. Box widths indicate group sizes proportional to √n, *n* = 498 (**A**). Comparison of EBOV-GP ELISA titre between Macenta samples in (**A**) and Guinean PCR-confirmed EVD survivors (*n* = 137) and their contacts (*n* = 90) sampled annually 2016-17. Note that the contact group includes a mix of symptomatic, asymptomatic and unaffected individuals (**B**). Centre = median. Box = interquartile range (ie. +/− 25% of the data either side of the median) i.e. 25th and 75th centiles. Whiskers = Q1 – 1.5*interquartile range and Q3 + 1.5* interquartile range.

Viable serum samples were collected from 498 of 499 participants and responses to EBOV-GP assessed on an indirect ELISA at 1 in 50 dilution. Total EBOV-GP IgG antibody ranged from 0.001 to 5.64 international units per millilitre (IUml^−1^). As our investigation focused on a single population with unknown exposure we classified EBOV-GP ELISA responses using latent profile analysis (LPA; Fig. 2). This identified three Gaussian-distributed populations of low (*n* = 206, geometric mean 6.9 × 10^−3^ IUml^−1^, 95% CI 6.6–7.3 × 10^−3^), intermediate (*n* = 278, geometric mean 0.025 IUml^−1^, 95% CI 0.023–0.027) and high antibody titre (*n* = 14, geometric mean 0.559 IUml^−1^, 95% CI 0.287–1.088).

We compared individual-level antibody responses from this population to those of longitudinally sampled (2016-17) PCR-confirmed EVD survivors using the same EBOV-GP ELISA (Fig. 2B). These findings show that both the high titre Macenta group and PCR-confirmed EVD survivor cohort demonstrate similar magnitude anti-EBOV-GP antibody levels. Figure 2B also plots titres from contacts of the EVD survivors. This group included asymptomatic, symptomatic and unaffected contacts though none were subjected to PCR-testing when symptomatic. Comparison with Macenta populations shows that both the low and intermediate titre groups span the lower range of anti-EBOV-GP responses observed in these contacts.

**Fig. 3:**
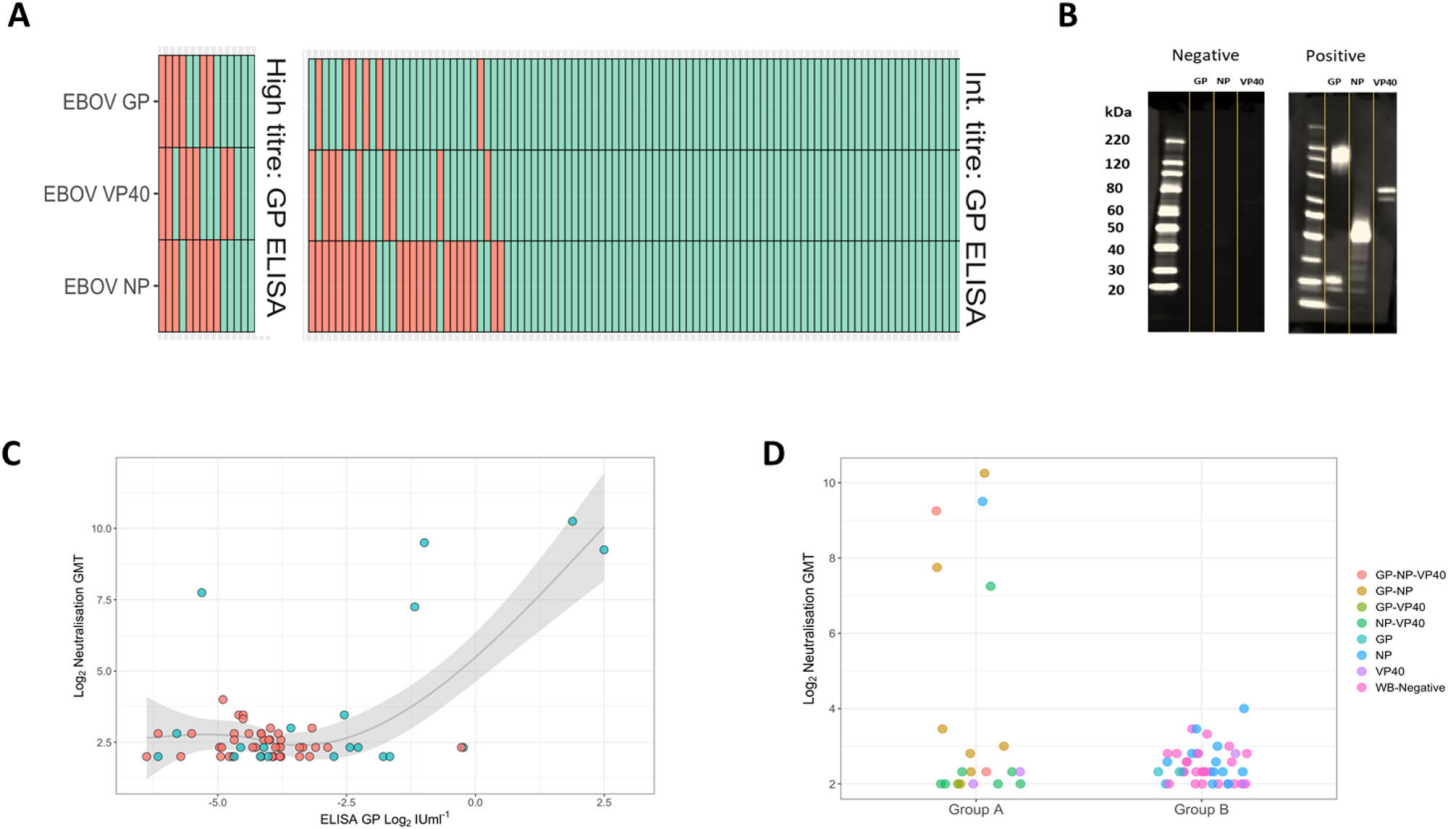
Analysis of serological responses of Macenta serum samples. Outcomes are stratified by high (left panel) and intermediate (right panel) titre EBOV-GP ELISA groups red dots indicate a positive result and green dots indicate a negative result (**A**). Representative western blots from a triple positive (JB154) and triple negative (JB050) sample, (**B**) Neutralisation titre of serum samples against EBOV strain Mayinga (*n* = 62; geometric mean titre of serum dilution) and regressed against paired ELISA-GP result using a cubic spline (*p* = 0.004 versus linear) blue dots indicate those samples that belong to latent class A and red dots indicate those samples that do not belong to latent class A (**C**) and stratified by latent class group blue points = group A, red points = group B. Grey shading denotes the estimated standard error of a natural spine with a maximum of 3 degrees of freedom. These are the results of a latent profile / finite mixture model, which classifies the most likely groups assuming they all form a Gaussian distribution in the total population. **D** Note the WHO EBOV international standard has an approximate value of ELISA GP IUml^−1^ Log2 of 2.5 and a Log_2_ neutralisation GMT of ~10.

To discriminate immunological responses among the Macenta samples, a sub-group of serum samples from intermediate (*n* = 101) and high (*n* = 14) EBOV-GP titre responses were selected for western blot (WB) analysis against 3 major antigenic EBOV proteins; glycoprotein (GP), nucleoprotein (NP) and matrix protein VP40. These data revealed complex individual-level responses (Fig. 3). The most common target binding was NP (31/115; 27.0%) followed by VP40 (16/115, 13.9%) and GP (12/115, 10.4%). Forty individuals demonstrated binding to at least one target (34.8%) whilst multi-target binding was observed among 17 individual serum samples (GP-NP-VP40 = 2/115, 1.7%; GP-NP 7/115, 6.1%; GP-VP40 1/115, 0.9%; GP-NP 7/115, 6.1%). Stratifying WB results by LPA classes, serum samples from individuals in both classes (group A and group B) recognized all 3 protein antigens (Fig. 3A). Importantly, the proportion of serum samples recognizing at least one protein antigen target by WB was greater among the high titre group (high: 11/14, 78.6%; intermediate: 29/101 28.7%, *p* = 0.0007).

Given these complex responses, we applied a latent class analysis to the results of the four combined assays, differentiating two classes of response (Supplementary Information 1.1). The first (group A: *n* = 20, median class probability = 92.2%) was characterised by a multi-target WB and high titre ELISA GP response. The second group (B: *n* = 95, median class probability = 98.4%) was defined by negative binding across all 3 WB protein antigen targets or some instances of single-target binding, mostly of NP, and an intermediate ELISA GP titre.

Serum neutralisation was then assessed from all participants of group A and sub-population of group B (42/95; 44%) using a well characterised live EBOV (strain Mayinga) assay. We identified 5/62 strong (range 1:152–1:1218 serum dilution), 5/62 low (range 1:10–1:16) and 52/62 absent ( <1:10) neutralisation responses. Neutralisation titre showed a bifurcated correlation with GP-ELISA titre (Fig. 3C) and all strong neutralisation responses occurred from sera classified within group A (Fig. 3D). A consort diagram outlining systematic sample selection process across all serological assays is provided in Supplementary Information (1.2).

**Fig. 4:**
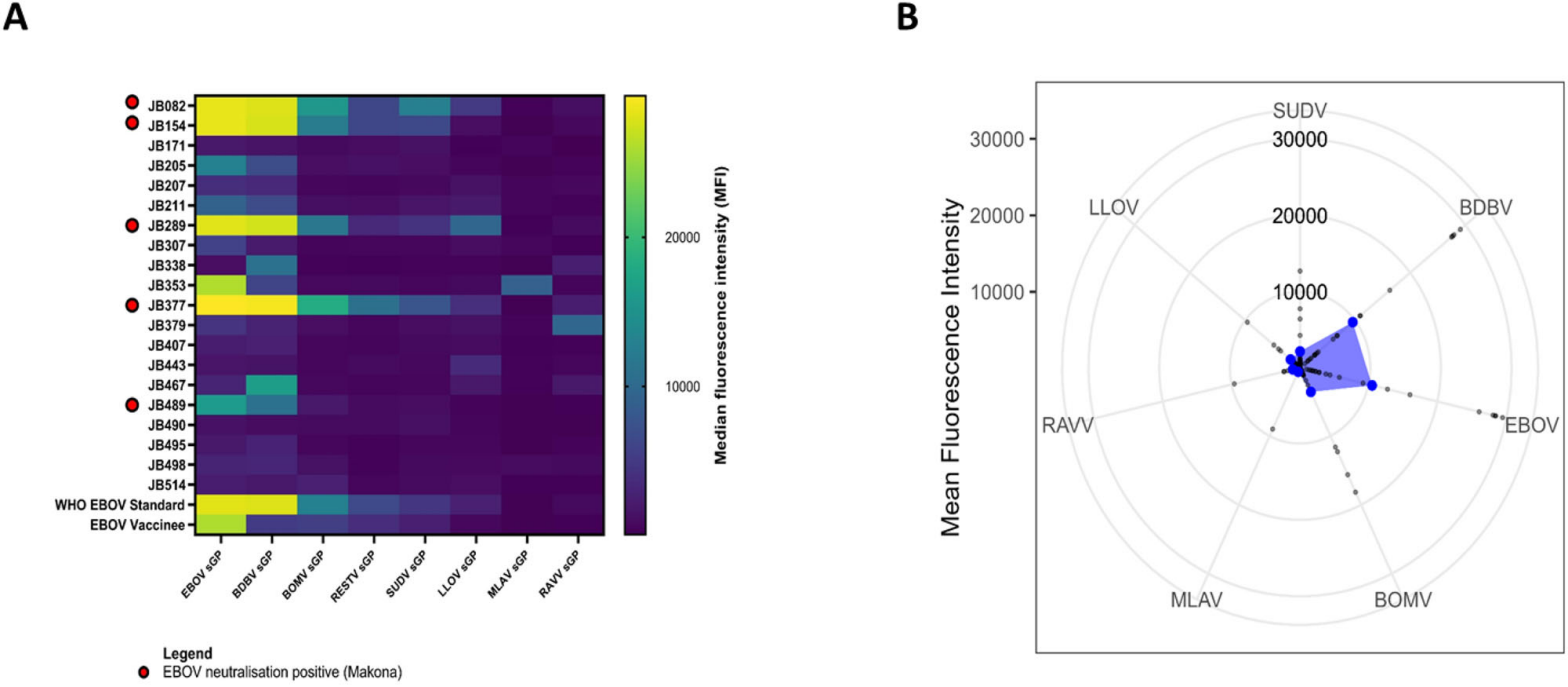
Broad serological analysis of Macenta serum samples. **A** Anti-filovirus total binding IgG antibody response as detected by Luminex-based multiplexed microsphere binding immunoassay for group A (*n* = 20). Median fluorescence intensity (MFI) values per glycoprotein (GP) antigen are shown for each individual (labelled by sample identification code). The World Health Organisation (WHO) Ebola Zaire (EBOV) standard and an ERVEBO® rVSV-ZEBOV vaccinee are included for comparison. **B** The average antigen-specific total binding IgG antibody response (median fluorescence intensity) for latent class group A against a multiplexed panel of filovirus antigens. Detected by Luminex-based multiplexed microsphere binding immunoassay.

Given the high degree of heterotypic cross-reactivity against the GP among the ebolaviruses, we next sought to examine whether other ebolaviruses may be implicated in these infections. All participants of latent class group A (*n* = 20) were further assessed for anti-filovirus GP antibody binding by multiplex microsphere-based immunoassay (MMIA). Total binding IgG antibodies against Ebola Zaire virus (EBOV), Bundibugyo virus (BDBV), Bombali virus (BOMV), Reston virus (RESTV), Sudan virus (SUDV), Lloviu virus (LLOV), Měnglà virus (MLAV) and Ravn virus (RAVV) GP were simultaneously measured for each sample. (Fig. 4).

All EBOV neutralisation positive samples (*n* = 5) showed the highest IgG against EBOV-GP but also strongly bound to BDBV GP, and further cross-reactions decreased proportionately with increasing phylogenetic distance from EBOV (Fig. 4A). The remainder (*n* = 15) showed no appreciable antibody response against any of the tested filovirus glycoproteins. We did not detect IgG binding to any of the GP of other ebolaviruses, e.g. TAFV or SUDV, at levels similar to that of EBOV GP. A sero-profile of group A highlighted that in these serum samples IgG preferentially bound to EBOV GP, and then to BDBV GP (Fig. 4B). This pattern of sero-reactivity is consistent with the notion that antigenic-relationships between the ebolaviruses closely follows phylogenetic relatedness. Individual serological profiles for each of the 20 participants in group A are provided in Supplementary Fig. 2.

### Epidemiology

**Fig. 5:**
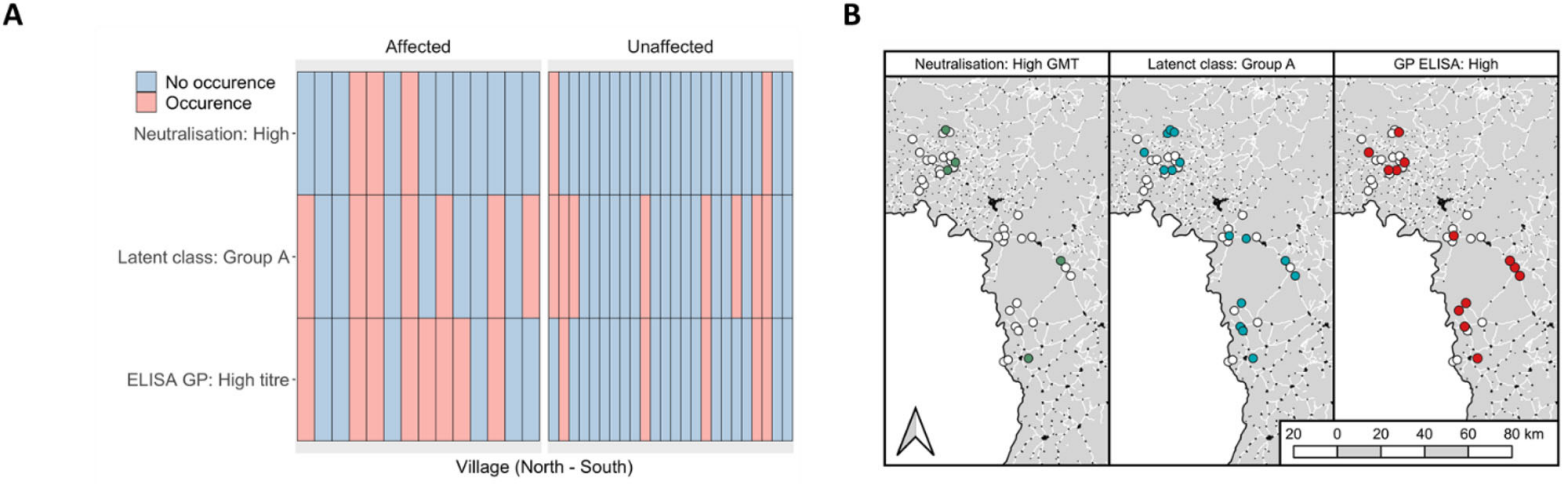
Spatial occurrence of serological outcomes. Heatmap of outcomes by village and further stratified by affected status. Note that participant numbers varied by village, so plots do not convey prevalence (**A**). Maps showing locations of sampled villages (white points) overlaid with occurrence of at least one serological outcome (**B**). **B** was generated using QGIS software.

We investigated potential mechanisms of exposure to EBOV and other filoviruses by comparing absolute location and spatial clustering of serological outcomes. To ensure comprehensive analysis, we separately examined high GP-ELISA (*n* = 14), group A (*n* = 20) and strong EBOV neutralisation (*n* = 5) as outcome measures. Occurrence of all three response types was spatially dispersed across the study sites with low numbers of seropositive individuals observed per village (Fig. 5). For high GP-ELISA and group A outcomes, we quantified spatial autocorrelation using Moran’s I on a series of model residuals (Table 1 and Supplementary Tables 3–5) with all outcomes exhibiting absence of residual spatial autocorrelation (Moran’s I range: −3.2 × 10^−3^ to 7.6 × 10^−3^). We next compared immunological responses by village status. All response types were identified across both affected and unaffected villages. Individuals with high GP-ELISA were more commonly found in affected villages although strong neutralisation and group A responses were more evenly distributed (Supplementary Table 2).

**Table 1:** Multivariable mixed-effects linear regression of log_2_ anti-EBOV-GP total antibody titre

Predictors	Estimate	95% CI	*p*-value
Village status			
Affected	Reference		0.13
Unaffected	−0.31	−0.70 to −0.08	
Age			
18–30	Reference		
31–50	0.33	0.04 to 0.63	0.08
51–90	0.30	−0.05 to 0.66	
Canopy cover (Closed forest)			
Shape index (500 m)	−0.50	−0.95 to −0.06	0.03
Vegetation			
(1000 m)	0.29	−0.07 to 0.64	0.12
Random Effects			
ICC	0.09		
N _village_	38		

*P*-values estimated by likelihood ratio 2-sided test.

Given the high-risk of zoonotic exposure to filoviruses, we next evaluated potential associations between local ecology and habitat disturbance with EBOV serological outcomes. Given the capacity of GP-ELISA to distinguish multi-target western blot responses and serum neutralisation, we modelled the association of anti-GP IgG antibody titre with a suite of demographic and environmental variables using a data-driven approach. This revealed an inverse association between the fragmentation of closed canopy forest and anti-GP titre (Table 1) following adjustment for village outbreak status (any confirmed EBOV case between 2013 and 16). The inverse relationship with closed forest fragmentation was consistent when using group A as a binomial model outcome (Supplementary Table 3). Further, the inverse relationship between closed canopy cover persisted when analysis was restricted to villages unaffected by the 2013–16 EBOV outbreak (Supplementary table 4). We also applied the same modelling approach with high GP-ELISA response as a binomial outcome and whilst the ecological scale and metric of fragmentation was different, the inverse relationship with fragmentation persisted (Supplementary Table 5).

## Discussion

We collected serum samples from a high-risk population including bushmeat hunters and their household contacts in Macenta Prefecture located within the forested region of Guinea. Samples were screened for immunological responses to EBOV using an objective, stepwise laboratory assessment incorporating a GP-specific screening ELISA, multi-target western blot analysis (GP-NP-VP40) and neutralisation assays using live EBOV. A notable proportion of this population (4.0%) demonstrated responses to multiple EBOV antigens (group A). This group included five individuals exhibiting clear EBOV Makona neutralisation and high IgG antibody titres against GP. Both these strong neutralising and other non-neutralising group A responses (*n* = 15) were observed in communities affected and unaffected by the 2013–16 EBOV outbreak. However, further epidemiological analysis failed to demonstrate overt demographic or spatial patterns in outcomes, though we observed a consistent inverse association between forest fragmentation and these distinct EBOV serological phenotypes.

We believe the multi-faceted serological signatures observed likely occurred from exposure to temporally and phylogenetically distinct pathogens. Among the five individuals exhibiting strong neutralisation responses, we observed strikingly similar serological phenotypes to those of PCR-confirmed Makona EVD survivors. We previously showed that 1-3 years post-infection, over 95% of EVD survivors exhibited persistent neutralisation, a finding corroborated by other longitudinal studies^18,22^. Further, our data indicate that GP-ELISA responses were closely correlated with neutralisation titre (Fig. 3), with concentration of the former comparable to titres exhibited by confirmed EVD survivors. Other studies using sera from EBOV-Makona survivors have shown high proportions ( >99%) exhibiting multi-antigen binding (GP, NP or VP40) as seen in all five individuals in our study^19^. These multi-antigen responses typically persist for several years after infection, reported as high as 96% in 2017 in a cross-sectional study, with greater waning to 66% during a 5-year longitudinal investigation. Interestingly, the five individuals reported here were distributed across communities previously affected (*n* = 3) and unaffected (*n* = 2) by EVD. However, resistance and under-reporting of EBOV cases was common in Guinée Forestière during the 2013–16 outbreak and it is plausible that unreported cases occurred in villages considered unaffected^12,23^. These participants may, therefore, represent previously undetected survivors of the 2013–16 outbreak. Given a prevalence of approximately 1% of this phenotype, this highlights the potential magnitude of under-reporting during the 2013–16 outbreak in rural areas of Guinée Forestière.

The other major sub-group we observed were individuals exhibiting antibody binding across multiple EBOV antigens in the absence of neutralisation, with these responses diverse in their combination of antigen targets and magnitude of binding. The aetiology of exposure among this group, therefore, appears less clear. Firstly, despite a low prevalence outcome, we do not consider assay specificity as influential given repeated binding of each sample across assays and different EBOV antigenic targets. Second, this group is unlikely to represent survivors of the 2013–16 outbreak. Whilst not all EBOV survivors generate neutralising antibody responses^24^, the vast majority do^18^ and the responses are persistent, and our sample collection occurred in 2017 when survivors would be expected to exhibit EBOV neutralisation. A third option is asymptomatic or mildly symptomatic survivors with different immunological phenotypes than those followed-up in large-scale cohorts in West Africa, largely recruited from Ebola Treatment Units (ETUs). Research in West Africa has established that asymptomatic EBOV infection can generate IgG antibody responses yet neither neutralisation nor the degree of protective immunity has been well-characterised. The incidence of asymptomatic EBOV-Makona infection resulting in detectable antibody responses is likely to range between 3 and 10% among contacts of cases^2,25^. If we putatively accept that all neutralisation responses we observed^5^ arose from symptomatic EBOV infection during the 2013–16 outbreak, the 15 non-neutralising multi-target responses occurred at an unfeasible ratio (5:15 or 75%) even after accounting for censoring due to EBOV mortality (typical case fatality rates of 56–78%^8,26^ equating to 11–21:15 or 42–57%). Thus, neither symptomatic nor asymptomatic infection during the 2013-16 outbreak can explain this phenomenon in totality.

For both groups we have described, recent or historical exposure to previously undetected outbreaks of EBOV or related filoviruses must be considered. This is particularly true for the non-neutralising cohort given a lack of feasible alternative hypotheses. In the region surrounding Macenta EBOV RNA fragments, BOMV complete genomes, and viable MARV were recently isolated from bats, representing multiple ecologically feasible pathways to spillover^6,10,12^. The first human Marburg virus case in West Africa was also isolated in a rural setting close to our study site, emphasising the unusually high-risk of zoonotic exposure to filoviruses faced by communities in this area. The epidemiological patterns we observed in our data also provide support for this zoonotic hypothesis. Firstly, the spatial occurrence of serological phenotypes was dispersed across the study area and is representative of expectations of zoonotic filovirus exposure under stuttering chain theory given the over-dispersed nature of EBOV transmission^27^. Our ecological modelling analysis also revealed a consistent inverse association of EBOV-directed immunological phenotypes with ecological fragmentation measures, specifically the proximity of intact closed canopy forest.

From an immunological perspective, the serological phenotypes we describe are also plausibly explained by historical exposure to EBOV or other non-EBOV filoviruses. In the few long-term follow-up studies of EBOV survivors, individuals exhibit persistent binding, even up to 40 years post-infection. Others have suggested that over a five-year period, persistence of GP-specific antibody responses is over 75% and 67% for at least two antigens^19^. Regarding the possibility of exposure to non-EBOV filoviruses, the GP of *Ebolaviruses* is highly cross-reactive between species although the magnitude of GP antibody binding varies between *Ebolavirus* species^17,28^. EBOV convalescent sera exhibits pan-species neutralisation though the proportion of individuals exhibiting cross-species neutralisation also varies dependent on infecting strain^29,30^. Cross-reactivity is not limited to the GP antigen but also occurs for both NP and VP40, including in Guinean survivors of Makona infection^19^. Within the wider filoviridae family, sera from MARV survivors does not typically neutralise ebolaviruses yet it can cross-react with certain ebolavirus antigens. Previous studies have identified a specific and temporally persistent affinity for EBOV-NP^31^ and a conserved, immunogenic GP domain shared by MARV and ebolaviruses^32^. Interestingly, in a sub-group of our cohort (group B) we observed multiple samples characterised by NP-only WB coupled to intermediate anti-GP titre.

We believe the objective, stepwise methodology we applied here to sample analysis was a major strength of our study and reinforces the need for similar approaches in future filovirus research. Stepwise approaches are particularly important given the evident ecological overlap and cross-reactivity of endemic filoviruses in West Africa. We also used objective classification methods to remove reliance on arbitrary cut-offs and control group samples acquired from unsuitable settings during analysis. Our study also has several limitations to consider. Due to sub-sampling from intermediate GP titre samples following ELISA screening, individuals who may have responded to subsequent WB and neutralisation assays may have been missed. As high titre GP-ELISA correlated well with responses on downstream assays, we do not expect these numbers to be substantial but the true number of individuals in group A may be higher than we present. Given purposive sampling of bushmeat hunters and their contacts in a limited geographical region, our findings are not generalisable but reflect exposure among one of the highest-risk populations in filovirus-endemic regions of West Africa. Given epidemiological links to similar groups in previous outbreaks, community engagement and surveillance among these populations must remain a priority. Disentangling exposure aetiology in Guinée Forestière where resistance to public health response was high and disease-related stigma persists is challenging. We have attempted to objectively consider plausible explanations and believe our results overcome some of these barriers to provide insight into filovirus exposure in these communities and identify targets for future surveillance.

This study was based on the hypothesis that, zoonotic spillover events of EBOV and related filoviruses, had occurred prior to and after the 2013–2016 outbreak in Guinea Forestière. Our comprehensive multi-staged serological analysis, ecological assessment and statistical analysis support this hypothesis. The various reports of filovirus detection in bats located in Guinea, Liberia and Sierra Leone, combined with the first MARV case close to our study site, provides logical support for our hypothesis. Numerous demographic, cultural and ecological factors need to align to result in a significant outbreak and most spillover events are likely restricted to a small number of cases which do not alert local health authorities yet leave an immunological footprint that can enable subsequent detection^27,33^. A major lesson learned from the ongoing COVID-19 pandemic is that the international community has paid little attention to the threat of emerging viruses with pathogen prioritisation exercises based on a lack of effective intelligence^34^. Our study further illustrates the need to combine serological, genomic and ecological evidence in development of risk-based approaches to identify areas most likely to give rise to outbreaks. It is not practical to keep a constant watch over the entire globe but focussing on high risk locations is certainly feasible.

## Methods

### Ethics

Ethical approval was obtained from the National Ethics Committee for Health Research, Guinea (No. 33/CNERS/15) and from the National Research Ethics Service, UK.

### Study setting and population

Macenta is a prefecture of Guinea located in the historically forested Nzérékoré region and borders Guéckédou prefecture, the index site of the 2013-16 EBOV epidemic and 2021 MARV outbreak. The population is mainly rural and dependent on an agricultural economy whilst hunting of sylvatic species is widely practised by specific families in rural settlements. Macenta has seen extensive deforestation historically with continuing tree cover loss of 26% between 2000 and 23^35^. Nzérékoré region has high levels of poverty (45.6% below the poverty line in 2018) and limited access to health services (1 doctor per 14,000) below WHO minimum recommended standards.

Between February-December 2017 Toma-speaking villages in Macenta were purposively selected to include those both affected and unaffected by the 2013-16 EBOV epidemic. All villages officially designated as EBOV unaffected underwent further screening by Toma-speaking staff prior to inclusion to exclude the possibility of undocumented cases, through key informant interviews of healthcare workers and village leaders. Following selection of villages, each hunting family was approached for participation. One household from each family was included, with the senior hunter and their spouse, or the next closest household relative, invited to participate. We also present comparative serological data from PCR-confirmed EBOV Makona survivors and their household contacts, sampled in Guéckédou and Coyah prefectures. These samples were collected longitudinally in 2017. This study was ethically approved by the board of UK research ethics council as well as the national research for health ethic committee of Guinea (permit N°012/CENRS/2017).

### Procedures

Consenting participants were invited to local health centres where 5 ml peripheral blood was collected following administration of a sociodemographic questionnaire. Blood samples were centrifuged at 2000 g for 10 min and serum aliquoted and stored at −20 °C.

### Enzyme linked-immunosorbent assay (ELISA)

ELISA was performed using a recombinant EBOV-GP protein, Makona strain (Oxford University, Nuffield Department of Medicine UK), for which Nunc Maxisorb 96 well plates were coated overnight (16–18 h) with purified EBOV-GP antigen (1 µg/ml). Plasma was serially diluted, starting at 1:50 in Casein (37528, Thermo Scientific, UK) and bound IgG detected using goat anti-human IgG Fcγ specific antibody conjugated to alkaline phosphatase (AP) (1:2000) (A3187, Sigma Aldrich). AP-Yellow substrate was added and the OD measured at 405 nm using a VERSAmax plate reader controlled by SoftMax Pro Enterprise software (V4.7.1). The plates were read using a predefined Softmax template which fits a 4-parameter logistic (4PL) curve to the dose response data.

### Western blot

Recombinant GP was sourced from Nuffield Department of Medicine, Oxford University, Oxford, UK and was based on the Makona strain of EBOV. Nucleoprotein (NP) was purchased from Gentaur (MBS1206629, partial expression 488-739) and is based on Myinga EBOV. VP40 was based on Myinga strain of EBOV and was purchased from Gentaur (IT-014-011Ep). Proteins were heat denatured and loaded onto 4–12% BisTris gels and separated by size by SDS-PAGE. The proteins were then transferred to PVDF membrane and blocked overnight in block buffer (PBST buffer with 5% milk (w/v)). Plasma was diluted 1:1000 in block buffer and incubated with the EBOV-protein containing blots for four hours at room temperature, then washed for five minutes in PBST. Secondary antibody; Anti-Human IgG (y-Chain Specific) peroxidase conjugate developed in goat; F(ab’)2 fragment (A2290, Sigma Aldrich, UK), was prepared at 1:1000 dilution in block buffer. The blots were incubated with secondary antibody for one hour at room temperature. Membranes were washed and blots developed with ECL prime (RPN2232, Sigma Aldrich, UK), incubating for five minutes. Images were captured at five and ten-minute exposure and presence of immunoreactivity determined against a molecular marker standard (LC5602, Life Technology, UK).

### Virus neutralisation assay

The activity of the EBOV-specific antibodies was determined by neutralisation of EBOV variant Mayinga (1976) as previously described in ref. ^36^. Briefly, in a biosafety level 4 (BSL4) laboratory and following heat treatment for complement inactivation, samples were serially diluted in supplemented Dulbecco’s modified Eagle’s medium (DMEM) in 96-well culture plates, 100 TCID_50_ units of EBOV variant Mayinga were added to the plasma dilutions. Following incubation at 37 °C for one hour, Vero cell suspension in supplemented DMEM was added. Plates were then incubated at 37 °C with 5% CO_2_ and cytopathic effects were evaluated at seven days post-infection. Neutralisation titres were calculated as Geometric Mean Titre (GMT) of four replicates.

### Statistical analysis

All statistical analysis was conducted in R version 4.0.4. Gaussian and non-gaussian serological outcomes were classified using latent profile (mclust version 5.4.7) and latent class procedures (poLCA version 1.4.1) respectively. To model the association between each serological outcome and local environment, we assembled a land cover classification scheme based on remote sensing imagery for 2017 at 100 m resolution. Land cover was defined as closed forest (canopy cover > 70%), open forest (cover 15–70%), shrubs, herbaceous and urban. We calculated land cover proportions and fragmentation indices (fractal dimension index, perimeter-area ratio, shape index) across a range of buffer distances (0.5–20 km) measured from the centroid of all sampled villages. We modelled extracted environmental data alongside sociodemographic data (village status, population, age, sex) against each serological outcome. We used mixed-effects generalised models with random intercepts allowed for villages where appropriate, with variables assessed for univariate association with each outcome measure. Due to collinearity across environmental data, for each model, a correlation matrix was developed inclusive of all variables exhibiting likelihood ratio *p*-values <=0.20 in univariate analysis, with one of each colinear pair (Pearson correlation coefficient >=0.75) removed based on comparative AIC value. Final models were fitted using a stepwise forwards AIC process and final models were assessed for model assumptions. Spatial dependency of model residuals was assessed using Moran’s I (spdep version 1.1–5).

### Multiplexed serological analysis by Luminex™

Serum samples were screened with the Luminex-based multiplex microsphere immunoassay (MMIA)^37–39^. Included in the protein antigen panel were GP of Ebola Zaire virus (EBOV), Bundibugyo virus (BDBV), Bombali virus (BOMV), Reston virus (RESTV), Sudan virus (SUDV), Lloviu virus (LLOV), Měnglà virus (MLAV) and Ravn virus (RAVV). Sera from the World Health Organization (WHO) EBOV serology standard, an ERVEBO® rVSV-ZEBOV vaccinee, and an Ebola Zaire-negative pool were also included for comparison.

Briefly, ectodomains of attachment GP were identified in Genbank, the transmembrane domain was removed, and a GCN3 motif that promotes trimeric protein folding was added to the C-terminus. Recombinant GP were cloned into a pcDNA3.1 expression vectors and transfected into FreeStyle 293-F cells (FreeStyle 293 Expression System, Thermo Fisher). Stable cell lines were selected and recombinant soluble ecotodomain GP were purified by size exclusion and affinity chromatography utilizing methods adapted for the expression and purification of henipavirus envelope glycoproteins^40^. Protein tags (S-tag or Twin-Step-tag) were cleaved after elution and purified GP were stored at −80 C prior to coupling to magnetic microspheres. Recombinant GP (30 ug) were coupled to 100 uL of magnetic microspheres (Luminex, Austin, Tx).

Samples were heat inactivated at 60 degrees Celsius for 30 min and diluted at 1:400 in PBS. Diluted serum samples were incubated with a master mix of the GP-coupled microspheres. GP bound antibodies were detected using biotinylated anti-human IgG (Thermofisher, United Kingdom), followed incubation with streptavidin-phycoerythrin (PE) for detection of biotinylated IgG (Bio-Rad). Antigen-antibody complexes were measured by a Bio-Plex 200 multiplexing system and with Bio-Plex Manager Software (Bio-Rad). Levels of GP bound IgG were reported as the median fluorescence intensity (MFI) of 100 microspheres counted per region; MFI is proportional to the amount of total IgG antibody bound to each GP.

Positivity thresholds for the Luminex serological assay have not been defined for this population due to the lack of negative and single-infection control human serum, and the extensive serological cross-reactivity between antigenically related filoviruses seen at the machine upper limits of quantification^38^.

### Reporting summary

Further information on research design is available in the Nature Portfolio Reporting Summary linked to this article.

### Supplementary information

Supplementary Information

Reporting Summary

## Data Availability

All data generated in this study have been deposited in FigShare 10.6084/m9.figshare.25635051.
