# Supplementary material for: Serological evidence of zoonotic filovirus exposure among bushmeat hunters in Guinea
Source: Nat Commun. 2024 May 16;15:4171. doi: 10.1038/s41467-024-48587-5 (PMC11099012; doi:10.1038/s41467-024-48587-5)
Supplement: Supplementary file 1 — Supplementary Information [file 41467_2024_48587_MOESM1_ESM.pdf]

Supplementary Information

1.1 Summary of latent class analysis output

Assay result	Assay +ve / Group N	Risk	Risk Ratio
**GP ELISA High Titre**			
Group A	11 / 20	0.550	17.4
Group B	3 / 95	0.032	(5.3 – 56.8)
**WB GP positive**			
Group A	10 / 20	0.500	23.7
Group B	2 / 95	0.021	(5.6 – 100.2)
**WB NP positive**			
Group A	17 / 20	0.850	5.8
Group B	14 / 95	0.147	(3.4 – 9.7)
**WB VP40 positive**			
Group A	4 / 20	0.042	14.2
Group B	12 / 95	0.600	(5.1 – 39.7)
**WB negative**			
Group A	0 / 20	0.789	-
Group B	75 / 95	0	

Supplementary table 1: Results of latent class analysis from GP-ELISA

1.2 Consort diagram of sample processing pipeline and selection process

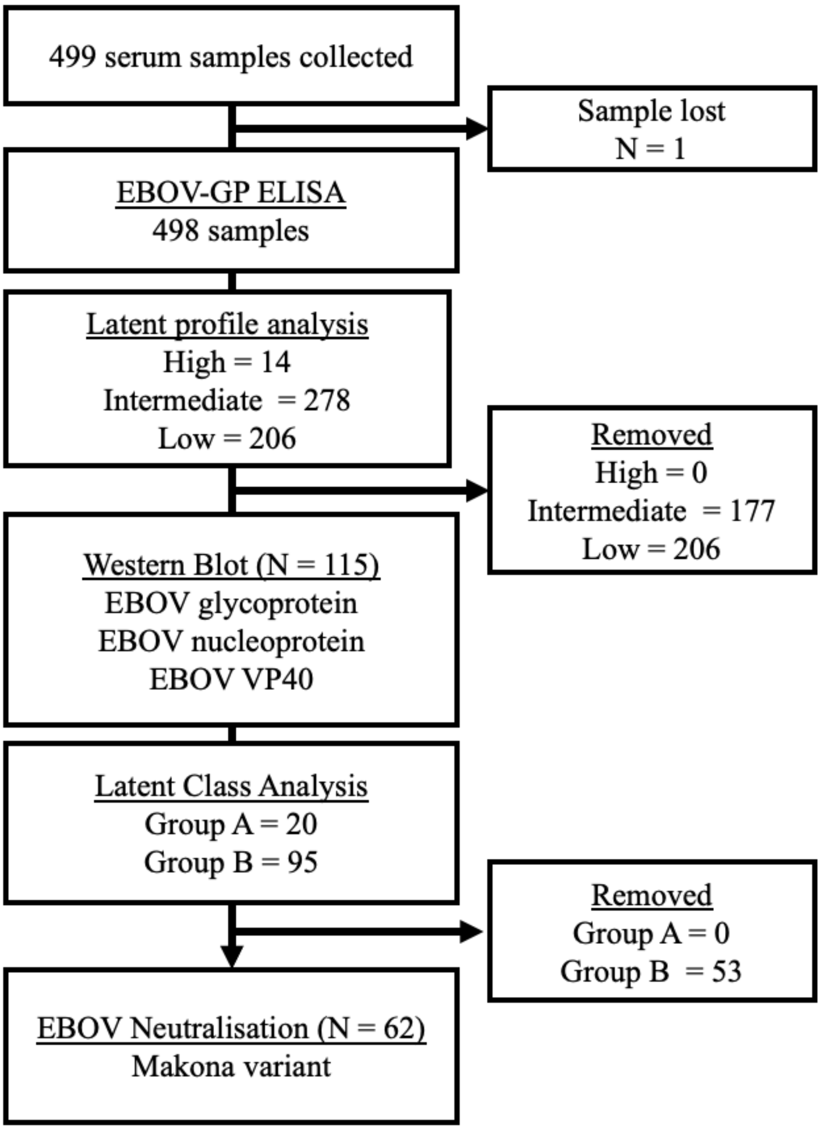

Supplementary figure 1: Consort diagram of serum sample processing.

1.3 Outcomes of serological analysis stratified by village status

	Affected (N=194)	Unaffected (N=304)	Overall (N=498)
**Anti-GP ELISA**			
High	9 (4.6%)	5 (1.6%)	14 (2.8%)
Intermediate	111 (57.2%)	167 (54.9%)	278 (55.8%)
Low	74 (38.1%)	132 (43.4%)	206 (41.4%)
**Latent class group**			
Group A	8 (16.3%)	12 (18.2%)	20 (17.4%)
Group B	41 (83.7%)	54 (81.8%)	95 (82.6%)
Not tested	145	238	383
**Neutralisation data**			
High	3 (10.3%)	2 (6.1%)	5 (1.0%)
Low	3 (10.3%)	2 (6.1%)	5 (1.0%)
Negative	23 (79.3%)	29 (6.1%)	52 (10.4%)
Not tested	165	271	436

Supplementary table 2: Serological outcome data stratified by village status. Villages were classified as affected or unaffected by 2013-2016 EBOV outbreak (see methods).

1.4 Ecological associations with EBOV immunological outcomes: Sensitivity analysis

Predictors	Odds ratio	95% CI	p-value
**Outcome**			
LCA group A	20 / 498		
**Village status**			
Affected	Reference		0.86
Unaffected	1.09	0.40 – 2.94	
**Age**			
	1.03	0.99 – 1.06	0.12
**Closed forest**			
Shape index (500m)	0.28	0.08 – 0.98	0.02
**Vegetation**			
Perimeter area ratio (20,000m)	0.35	0.08 – 0.98	0.01
**Random Effects**			
ICC	0.02		
$N_{village}$	38		

Supplementary table 3: Multivariable generalised linear mixed effects model (binomial family) of immunological group defined by latent class analysis of ELISA and Western Blot analysis (Group A vs Group B). Success defined as Group A. Variables were selected using a forward, stepwise approach using AIC. P-values estimated by likelihood ratio test. Mixed effect models not used due to singular fit from village-level random intercepts. Two-sided test.

Predictors	Estimate	95% CI	p-value
**Age**			
18 – 30	Reference		
31 – 50	0.19	-0.15 – 0.53	0.55
51 – 90	0.15	-0.25– 0.55	
**Closed canopy cover**			
Perimeter area ratio (500m)	-0.63	-1.24 – -0.02	0.05
**Random Effects**			
ICC	0.14		
$N_{village}$	24		

Supplementary table 4: Multivariable mixed-effects linear regression of log_2 anti-EBOV-GP total antibody titre excluding all participants from villages with confirmed EBOV cases during 2013-2016 outbreak (195/498; 39.2%). Variables were selected using a forward, stepwise approach using AIC. P-values estimated by likelihood ratio test. Two-sided test.

Predictors	Odds ratio	95% CI	p-value
**Outcome**			
High titre GP-ELISA	14/498		
**Village status**			
Affected	Reference		0.24
Unaffected	0.35	0.14 – 1.56	
**Age**			
	1.02	0.98 – 1.06	0.35
**Vegetation**			
Perimeter area ratio (20,000m)	0.37	0.12 – 1.04	0.06

Supplementary table 5: Multivariable generalised linear model (binomial family) of log_2 anti-EBOV-GP total antibody titre classified by finite mixture models (high titre individuals vs. intermediate and low titre individuals combined; see figure 1). Success defined as high titre individual. Variables were selected using a forward, stepwise approach using AIC. P-values estimated by likelihood ratio test. Two-sided test.

Supplementary Figure 2

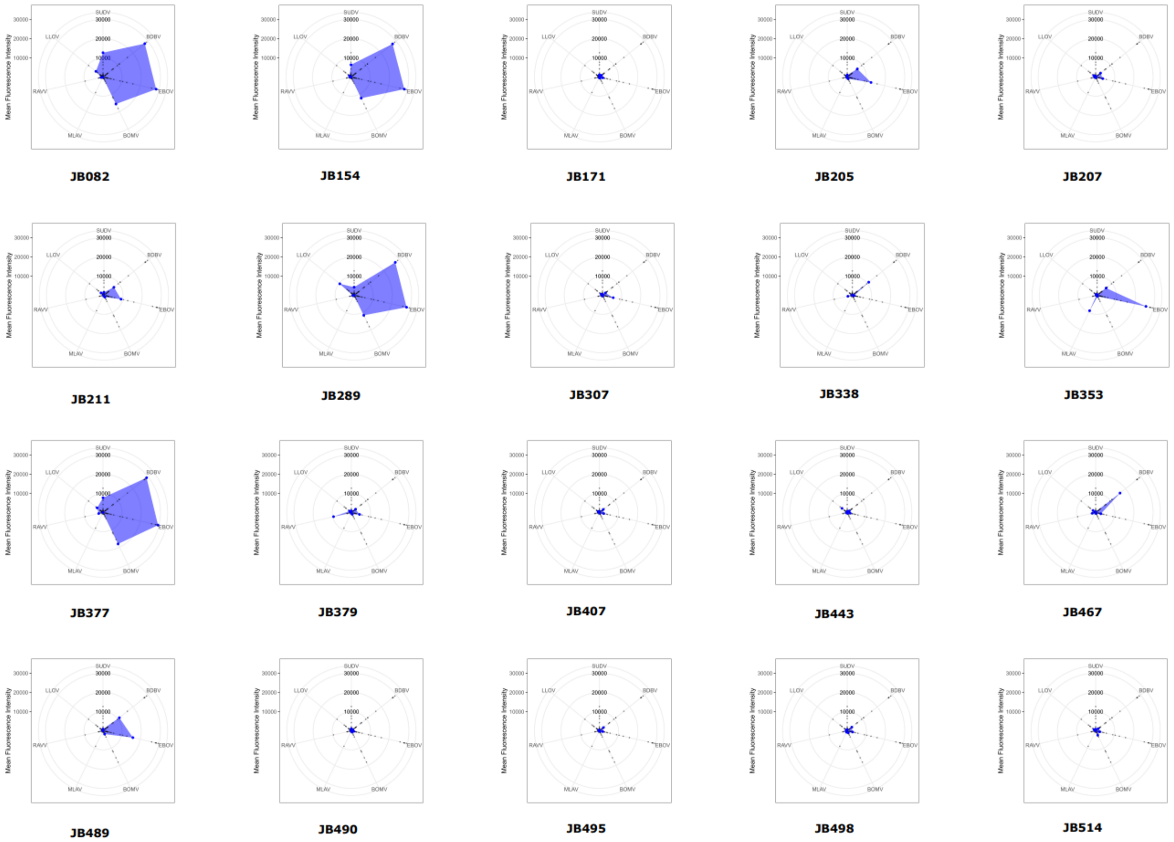

Individual serological profile of participants within group A (n=20). Shows antigen-specific total binding IgG antibody response (median fluorescence intensity) against a multiplexed panel of filovirus antigens. Detected by Luminex-based multiplexed microsphere binding immunoassay
